# Baseline and Follow-Up Cardiac Magnetic Resonance Imaging Findings in Children with Acute Myocarditis and Factors Associated with Late Gadolinium Enhancement

**DOI:** 10.3390/jcm14010189

**Published:** 2024-12-31

**Authors:** Bekir Yukcu, Merve Maze Aydemir, Mehmet Balci, Mehmet Kanyilmaz, Aysel Turkvatan, Selman Gokalp, Alper Guzeltas, Sezen Ugan Atik

**Affiliations:** 1 Department of Pediatric Cardiology, Istanbul Mehmet Akif Ersoy Thoracic and Cardiovascular Surgery Training and Research Hospital, University of Health Sciences, Istanbul 34303, Turkey; maze_zabun@hotmail.com (M.M.A.); balci67@gmail.com (M.B.); selmangokalp@gmail.com (S.G.); alperguzeltas@hotmail.com (A.G.); sezenugan@hotmail.com (S.U.A.); 2 Department of Radiology, Istanbul Mehmet Akif Ersoy Thoracic and Cardiovascular Surgery Training and Research Hospital, University of Health Sciences, Istanbul 34303, Turkey; drkanyilmaz@gmail.com (M.K.); aturkvatan@yahoo.com (A.T.)

**Keywords:** cardiac magnetic resonance imaging, children, heart, Lake Louise criteria, myocarditis, T1 mapping, T2 mapping

## Abstract

**Objectives**: Cardiac magnetic resonance (CMR) plays a central role in the diagnosis and follow-up of acute myocarditis (AM). In this study, we aimed to evaluate baseline and follow-up CMR findings and associated factors in children with AM. **Methods**: A retrospective analysis of CMR in pediatric patients with clinical presentations suggestive of myocarditis was performed. Patients’ demographic characteristics, clinical data, and diagnostic test results, as well as CMR imaging results, were evaluated. **Results**: All 28 pediatric patients with acute myocarditis included in this study had late gadolinium enhancement (LGE) on initial CMR imaging. Additionally, 14 (50%) patients had increased extracellular volume (ECV), 4 (50%) patients had focal high-intensity areas on T2 STIR images, 15 (53.6%) patients had increased T1 relaxation time, and 17 (60.7%) patients had increased T2 relaxation time. At a median follow-up CMR of 6 months, 24 (85.7%) patients had LGE, 5 (17.9%) patients had increased ECV, and 7 (25%) patients had increased T1 relaxation time, while other parameters showed complete recovery. Baseline troponin and CRP levels, T1 relaxation time, T2 relaxation time, and increased ECV were found to be factors associated with the resolution of LGE. **Conclusions**: Baseline troponin and CRP levels, as well as T1 relaxation time, T2 relaxation time, and increased ECV, were effective parameters that seemed to predict the resolution of LGE. Larger and multicenter experiences would confirm these hypotheses.

## 1. Introduction

Myocarditis is a pathological condition resulting from either acute or chronic inflammation of cardiac myocytes. This leads to associated myocardial edema and subsequent myocardial injury or necrosis [1]. The precise incidence of this condition remains elusive, but it is likely to be significantly underestimated, as many mild cases may be asymptomatic or present with minimal non-specific symptoms [2]. Numerous studies have indicated that males have a higher diagnosis of myocarditis than females, with an estimated incidence ranging from 0.80 to 2.13 cases per 100,000 individuals [3]. The diagnosis and subsequent management of pediatric myocarditis are of utmost importance. AM continues to be acknowledged as a potential cause of sudden cardiac death in both children and athletes, with incidences reported as high as 12% [4,5,6].

In children, myocarditis is most often caused by a viral or infectious etiology, such as parvovirus-19 and human herpesvirus 6 [7]. Other causes of myocarditis include autoimmune, toxins, drugs, and hypersensitivity reactions [3]. The clinical manifestation exhibits considerable variability, encompassing a spectrum from asymptomatic cases to those presenting with critical illness in the pediatric population. The predominant baseline symptoms comprise fever and a viral prodrome, which is observed in a minimum of 50% of the affected individuals [8]. Approximately two-thirds of the cohort presents with these symptoms. Tachypnea and gastrointestinal manifestations, such as nausea accompanied by vomiting and abdominal discomfort, are also frequently encountered among children. Additional prevalent presentations include thoracic pain, palpitations, dyspnea, exercise intolerance, and syncope [9,10]. More severe presenting symptoms encompass profound heart failure, ventricular arrhythmias, and instances of sudden cardiac death attributable to arrhythmias [11,12].

The diagnosis of myocarditis is made after a comprehensive evaluation of the patient’s history, clinical symptoms, laboratory parameters, electrocardiography, and imaging techniques. Since respiratory syncytial virus, COVID-19, and influenza viruses cause myocarditis, it is very important to take nasal swabs in clinical practice in cases where myocarditis is suspected [7]. Endomyocardial biopsy is considered the gold standard for definitive diagnosis of myocarditis but is often discouraged in children due to its invasive nature and difficulty in obtaining accurate samples [3]. Cardiovascular magnetic resonance (CMR) imaging has been shown to accurately depict AM without the need for invasive intervention [13]. CMR allows the characterization of the entire myocardium and accurate assessment of ventricular volumes and ventricular function in children with clinically suspected acute myocarditis [14,15]. The Lake Louise Criteria (LLC) is the traditional guideline for diagnosing myocarditis by CMR by evaluating the presence of hyperemia, edema, and myocardial necrosis/fibrosis [16]. The LLC sensitivity for diagnosing myocarditis is up to 87.5%, and specificity is up to 96.2% [17].

The prognosis of myocarditis depends on the change in clinical and functional parameters and the response to medical treatment. Determining prognostic factors is very important for identifying patients at higher risk of myocarditis. CMR is used both in the diagnosis and follow-up of myocarditis. Therefore, this study aimed to identify predictors of CMR associated with worse cardiovascular outcomes in myocarditis.

## 2. Materials and Methods

This retrospective study was conducted between 31 October 2019 and 31 December 2022 in patients who underwent CMR with a preliminary diagnosis of AM at the Istanbul Mehmet Akif Ersoy Thoracic and Cardiovascular Surgery Training and Research Hospital. Inclusion criteria were patients under the age of 18 who had a clinical presentation suggesting myocarditis that was later confirmed by CMR. Exclusion criteria were patients who did not receive CMR at baseline or whose follow-up CMR data were not available. Patients who were diagnosed with myocarditis after COVID-19 vaccination were also excluded. The files of 127 patients diagnosed with AM within the specified date range were reviewed, and 28 patients who met the inclusion criteria were enrolled in this study. Informed consent for participation was obtained from all subjects involved in this study.

The clinical diagnostic criteria hinged on symptoms such as acute chest pain, indicators of acute myocardial injury (changes in electrocardiogram [ECG] and/or elevated troponin levels), and signs consistent with infectious etiology (increased leukocyte count and/or C-reactive protein and/or verified infectious disease). Clinical presentations, including signs and symptoms, as well as diagnostic test results (C-reactive protein [CRP], procalcitonin, CK-MB, and troponin T with a normal value of <34 ng/L) and administered medical treatments, were documented. Echocardiographic evaluations encompassing the left ventricular ejection fraction (LVEF) and shortening fraction were performed upon admission and during subsequent follow-up visits. Experienced pediatric cardiologists executed two-dimensional standard echocardiography using the Philips EPIQ 7C system (Philips Healthcare Royal Philips Electronics, Amsterdam, The Netherlands).

### 2.1. CMR Acquisition Protocol

CMR was performed with a 1.5 Tesla MR scanner (Magnetom Aera; Siemens Healthcare, Erlangen, Germany) using dedicated cardiac software, a phased-array surface receiver coil, and vector-cardiographic triggering. Cine images were acquired using a breath-hold balanced steady-state free precession sequence (b-SSFP) in long-axis (2-chamber, 3-chamber, and 4-chamber) and short-axis views (6–8 mm slices without gap, 10–15 slices). T2-weighted MR imaging (short-tau inversion recovery [STIR] T2-weighted sequence) and T2 mapping were used to detect myocardial edema. For pre-contrast T1 mapping, we used the ECG-triggered modified Look-Locker inversion recovery (MOLLI) sequence (using scheme 3(3)3(3)5) on three short-axis (basal, mid, and apical) LV slices. Ten minutes after administration of a 0.15 mmol/kg intravenous bolus of gadobutrol (Gadovist, Bayer Healthcare, Berlin, Germany), LGE images were acquired using a 2D breath-hold phase-sensitive segmented inversion-recovery gradient echo pulse sequence in the same orientations as the cine images. Inversion time was individually optimized to null normal myocardium. Post-contrast T1-mapping was acquired following LGE imaging (typically 20 min after gadobutrol bolus injection) using a MOLLI sequence (4(1)3(1)2 scheme).

The aforementioned CMR evaluation was repeated for all patients six months later. In collaboration with a radiologist with experience in CMR, a multidisciplinary team with expertise in myocarditis reviewed the patient outcomes.

### 2.2. Image Analysis

All CMR studies were analyzed offline using a workstation with dedicated cardiac software (Syngo.Via software version 20A, Siemens Healthineers, Erlangen, Germany), and a consensus was reached between two blinded radiologists experienced in CMR. The presence of myocardial LGE was visually evaluated, and its extent was semi-quantitatively reported according to the American Heart Association’s 17-segment model [18]. AM was diagnosed according to Lake Louise criteria [13,19,20]. The reference values for T1 and T2 mapping used in this study were based on the latest literature, with normal T1 values ranging from 950 to 1050 ms and T2 values ranging from 45 to 55 ms [21].

This study followed the Lake Louise Criteria, revised in 2018, to diagnose myocardial inflammation in cases of suspected myocarditis. These criteria specify that cardiac magnetic resonance (CMR) findings are consistent with myocardial inflammation if at least two of the following are present: (1) Increased regional or global myocardial signal intensity on T2-weighted images, indicating myocardial edema, (2) Elevated global myocardial early gadolinium enhancement (EGE) ratio between myocardium and skeletal muscle in gadolinium-enhanced T1-weighted images, reflecting myocardial hyperemia and inflammation, (3) Presence of at least one focal lesion with non-ischemic regional distribution in inversion recovery-prepared gadolinium-enhanced T1-weighted images (late gadolinium enhancement, LGE), representing irreversible myocardial necrosis or fibrosis. In addition to these primary criteria, we also considered supportive criteria for further assessment and evaluation: (a) Pericardial inflammation, evidenced by pericardial effusion or signal intensity increase. (b) Left ventricular dysfunction, identified as systolic LV wall motion abnormalities.

### 2.3. Statistical Analysis

SPSS Statistics (version 25; IBM Corp., Armonk, NY, USA) was used for the statistical analysis. The Shapiro–Wilk test was used to assess the normality of each variable. Categorical variables were reported as absolute numbers and percentages. Continuous variables were expressed as means and standard deviations (SD) or as medians, if not normally distributed. The Mann–Whitney U test was applied for continuous non-parametric variables, and the chi-square test or Fisher’s exact test was used for categorical variables to determine the presence of statistically significant differences between groups. To compare the baseline and follow-up cardiac MRI parameters of the patients, the non-parametric paired test, Wilcoxon Signed Ranks Test, was used. Correlation analyses were conducted using Spearman’s rank correlation coefficient, with a predefined level of statistical significance at *p* < 0.05.

## 3. Results

### 3.1. Patient Characteristics

Demographic and clinical data of the patients are presented in Table 1. There were 27 males (96.4%) and 1 female (3.6%). The median age was 16 (min–max: 9–18 years). The mean weight and height of the patients were 65.6 kg (±15.2 kg) and 169 cm (±9.2 cm), respectively. The most common presenting complaints were chest pain in 26 (92.9%) patients and fatigue in 11 (39.3%) patients. While the electrocardiogram of 14 (50%) patients was normal, 7 (25%) patients had ST elevation, and 2 (7.1%) patients had ST depression. Two patients had pericardial effusion, two patients had a shortening fraction < 28%, and four patients had a positive viral panel.

### 3.2. CMR Imaging Results

**Figure 1:**
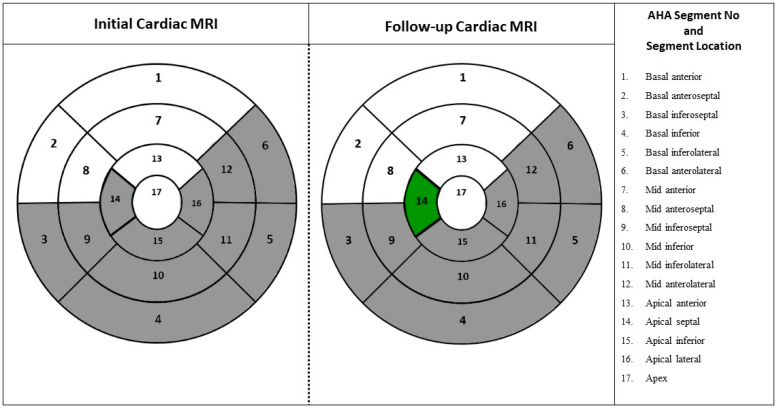
Distribution of LGE in AHA myocardial segments: This figure illustrates that patients with LGE involvement in specific myocardial segments during baseline and follow-up cardiac MR examinations. Color descriptions: Gray: LGE involvement positive, White: LGE involvement negative, Green: Segments that show LGE involvement in the first CMR but no involvement in the second CMR. (Baseline MRI LGE involvement by segment: Segment no 3: 7.1% (n = 2), Segment no 4: 39% (n = 11), Segment no 5: 78.6% (n = 22), Segment no 6: 14.3% (n = 4), Segment no 9: 21.4% (n = 6), Segment no 10: 39.3% (n = 11), Segment no 11: 78.6% (n = 22), Segment no 12: 28.6% (n = 8), Segment no 14: 7.1% (n = 2), Segment no 15: 14.3% (n = 4), Segment no 16: 35.7% (n = 10), Follow-up MRI LGE involvement by segment: Segment no 3: 7.1% (n = 2), Segment no 4: 21.4% (n = 6), Segment no 5: 67.9% (n = 19), Segment no 6: 10.7% (n = 3), Segment no 9: 14.3% (n = 4), Segment no 10: 14.3% (n = 4), Segment no 11: 46.4% (n = 13), Segment no 12: 14.3% (n = 4), Segment no 15: 3.6% (n = 1), Segment no 16: 3.6% (n = 1)). **Abbreviations**: AHA: American Heart Association, MRI: Magnetic resonance imaging.

CMR results of the patients are given in Table 2. In the baseline CMR evaluations of the patients, LGE was detected in 28 patients, representing a 100% prevalence. When the number of affected segments was assessed, the median involvement was found to be 3 segments (1–6 segments). The most common involvement was observed in the mid-inferolateral (78.6%) and basal inferolateral (78.6%) segments (Figure 1). Furthermore, in the first CMR scans, an increase in extracellular volume (ECV) was detected in 14 patients (50%). The median percentage increase in ECV was 25% (20–45%). Focal high-intensity areas on T2 STIR images were identified in 14 (50%) patients. The T1 relaxation time, a marker of acute myocardial injury, was elevated in 15 patients (53.6%), with a median T1 relaxation time of 1058 ms (916–1158 ms). Additionally, T2 relaxation time, an indicator of diffuse myocardial edema, increased in 17 patients (60.7%), with a median T2 relaxation time of 55.5 ms (42–62 ms). Pericardial effusion was present in 4 patients (14.3%) and reduced LVEF (<55%) was observed in 4 patients (14.3%).

All patients underwent control CMR at 4–12 months (median 6 months, range 4–27 months). The second CMR revealed persistent LGE in 24 patients (85.7%); in 21 patients (75%), the number of affected segments had declined. Complete elimination of LGE was observed in 4 patients (14.3%), no change was noted in 2 patients (7.1%), and an increase was observed in 1 patient (3.6%). Decreased ejection fraction was observed in 2 patients (7.1%).

When comparing patients with persistent LGE and those with complete resolution on the second CMR, we found that baseline troponin-T levels, CRP levels, and T1 and T2 relaxation times, as well as ECV percentages in the baseline CMR, were significantly higher (*p* < 0.05) in the former group. However, there was no statistically significant difference between the two groups in terms of the number of segments with LGE on the baseline CMR and LVEF percentages (Table 3).

**Figure 2:**
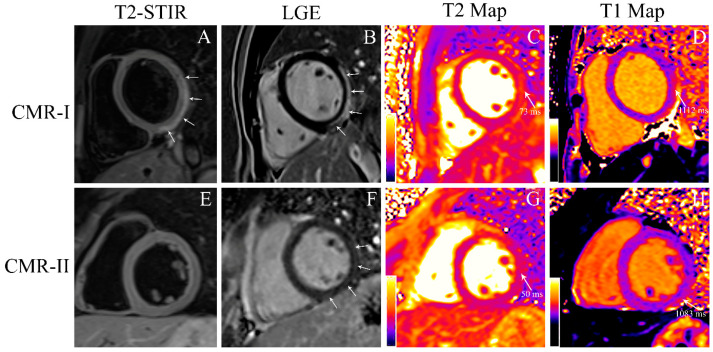
A 17-year-old male patient presented with acute chest pain. CMR was performed within the first week following symptom onset (CMR-I) and after 6 months (CMR-II). In the CMR-I images; T2-w STIR image (**A**) shows a hyperintense subepicardial rim (arrows) representing myocardial edema in the basal inferolateral and inferior segments of the left ventricle. LGE image (**B**) shows an increase in gadolinium uptake (arrows) in the same segments representing myocardial necrosis and acute extracellular edema. T2 map (**C**) and T1 map (**D**) images confirm the findings of acute myocardial inflammation: T2 mapping value is higher than 55 ms; native T1 value is higher than 1050 ms. In the CMR-II images; no edema is seen on T2-w STIR image (**E**) and T2 map image (**G**). T2 mapping value is lower than 55 ms. LGE image (**F**) shows increase of gadolinium uptake (arrows) representing fibrosis. T1 map (**H**) image confirms the findings of fibrosis; nativ T1 value is higher than 1050 ms. **Abbreviations**: LGE: late gadolinium enhancement.

Patients with increased T1 relaxation time had higher procalcitonin levels (*p* = 0.024) and troponin-T normalization time (0.043) than patients with unchanged T1 relaxation time. Patients with increased T2 relaxation time had higher troponin-T (*p* = 0.011) and procalcitonin levels (*p* = 0.021) than patients with unchanged T2 relaxation time. Patients with increased ECV had higher troponin-T (*p* = 0.020), CK-MB levels (*p* = 0.039), and troponin-T normalization time (*p* = 0.041) than patients with unchanged ECV (Table 4, Figure 2).

## 4. Discussion

This retrospective study evaluated the CMR results of patients with acute myocarditis. In the baseline CMR evaluations, 28 (100%) patients had LGE, 14 (50%) patients had increased ECV, 4 (50%) patients had focal high-intensity areas on T2 STIR images, 15 (53.6%) patients had increased T1 relaxation time, and 17 (60.7%) patients had increased T2 relaxation time. In the follow-up CMR, 24 (85.7%) patients had LGE, 5 (17.9%) patients had increased ECV, and 7 (25%) patients had increased T1 relaxation time, while other parameters showed complete recovery. It was determined that baseline troponin and CRP levels, as well as T1 relaxation time, T2 relaxation time, and increased ECV, were effective parameters in the resolution of LGE. Additionally, serum troponin-T, procalcitonin, and CK-MB levels were associated with increased T1 relaxation time, increased T2 relaxation time, and increased ECV.

In recent years, CMR imaging, which has been increasingly used, has established itself as the gold standard non-invasive test for the diagnosis of acute myocarditis [22]. The use of T1 and T2 mapping, as well as extracellular volume fraction, for diagnosing heart conditions is well-known in adults. However, there is a lack of similar research focusing on pediatric myocarditis. The established cutoff values for these mapping parameters in acute myocarditis vary widely due to differences in technical aspects, such as scanning equipment, software, and analysis methods. Additionally, age, gender, heart rate, and hydration status can affect T1 and T2 relaxation times [23]. Isaak et al. studied a pediatric population and showed that patients with reduced LVEF had elevated T1 and T2 values [23]. Although there were no significant variations in ECV fraction, LVEF, or troponin levels compared to patients who did not require intermediate care unit treatment, those who needed treatment in the intermediate care unit showed higher T1 and T2 relaxation times. In another study comparing children with AM and healthy controls, it was determined that children with acute myocarditis had a higher T2 ratio, early gadolinium enhancement ratio (EGEr), T1 relaxation times, T2 relaxation times, and extracellular volume fraction compared to controls [24]. In our study, baseline CMR assessments revealed higher T1 relaxation times in 53.6%, higher T2 relaxation times in 60.7%, and elevated ECV fraction in 50% of the cases. No significant relationship existed between reduced LVEF and the need for intensive care unit admission. However, in the three patient groups with high T1 and T2 relaxation times and ECV values, it took longer for troponin T levels to return to normal compared to those in whom these elevations were not observed. These findings have led us to suspect that elevated mapping results may indicate the severity of myocarditis.

The presence of LGE on CMR imaging in AM is an important finding. There is a need to develop a consensus regarding the timing for a follow-up CMR study in patients with AM if we use LGE as a marker for prognostic significance and risk stratification [25,26]. Yang et al. [22] conducted a thorough meta-analysis to show that the presence of LGE is a predictor of some unfavorable outcomes, including cardiovascular mortality, all-cause mortality, the need for a heart transplant, readmissions to the hospital, recurrent AM, and the need for mechanical circulatory support. In Lota et al.’s [27] study with adults, although the risk of cardiac death did not increase in the presence of LGE with normal LVEF, palpitations due to non-sustained ventricular tachycardia, cerebrovascular accident, pacemaker implantation, and atrial fibrillation were more frequent. Therefore, researchers have conducted studies to determine the change in LGE during follow-up with CMR. In some studies, evaluating the rate of change in LGE with control CMR at third or sixth months, resolution of LGE was reported in 27–49% of patients [28,29,30]. In their study evaluating 33 patients with AM, Pieroni et al. [31] determined LGE in 94% (31/33) of the baseline CMR. They observed a decrease in LGE (79%) in the third month of follow-up CMR with a decrease in edema. However, they observed that the CMR findings in the twelfth month were no different from the third month, and LGE persisted in 26 (79%) patients. Luetkens et al. [24], in their study, observed LGE in 91% (22/24) of the baseline CMR and in 67% (16/24) of the follow-up CMR after eight weeks. In a study of 52 children with viral or idiopathic myocarditis, LGE was found in 81% of patients on baseline CMR. After a median follow-up of 6 months, LGE was found in 83% of patients (75% persistent, 8% new) [14]. In their study of 18 adolescent patients with myocarditis, Małek et al. [32] observed the presence of LGE in all patients at the time of baseline CMR evaluation. Subsequently, in a follow-up CMR conducted at a median interval of 7 months, they noted that LGE persisted in 72% of cases. Our study observed similar trends, with 100% of patients exhibiting LGE during the baseline CMR and 85.7% still showing LGE in the follow-up CMR, which occurred at a median of 6 months after the baseline assessment. Our and previous study results suggest that LGE identified on baseline CMR in patients with AM largely persists and does not resolve.

The persistence of LGE on CMR indicates fibrosis and myocardial scarring. This condition predisposes to long-term cardiac risk in myocarditis [22,33,34]. Mewton et al. [35] showed that LGE is an independent predictor of cardiovascular events in AM, even after adjusting for age, LVEF, and clinical presentation category. Grun et al. [36] elucidated that the existence of LGE observed in baseline CMR imaging serves as the most robust independent prognostic indicator for overall mortality as well as cardiac-specific mortality, irrespective of the clinical manifestations present. Therefore, determining the risk factors associated with persistent LGE is vital for the follow-up of high-risk patients. Ferrero et al. [37] showed that QRS fragmentation on electrocardiography in acute myocarditis is associated with the presence and location of LGE. Berg et al. [28] documented that baseline cardiac enzymes and inflammatory markers did not predict change in LGE at 3 months. However, in our study, higher T1 and T2 relaxation times and increased ECV were found during baseline CMR in patients with persistent LGE compared to patients with resolved LGE. Moreover, higher troponin and CRP levels were observed at admission in the persistent LGE group compared to the LGE-resolved group. In addition, higher T1 and T2 relaxation times and increased ECV during baseline CMR were found to be associated with troponin-T, procalcitonin, and CK-MB. In myocarditis, higher troponin levels are associated with the severity of the disease and the degree of inflammatory involvement [38]. Therefore, the high troponin and CRP levels in our study suggest that the severity of inflammation at admission may affect LGE persistence. When the follow-up CMR of patients with LGE was compared with their baseline CMR, it was seen that the LGE percentage value decreased by 75%, remained unchanged at 7.1%, and increased by 3.6%. However, when these subgroups were evaluated according to age, CRP, procalcitonin, baseline troponin T, and the number of LGE segments in the baseline CMR, these value differences were not statistically significant.

The strength of our study is that it reveals the factors associated with persistent LGE. However, it also has some limitations. The first limitation is the retrospective design and the related information bias. The second limitation is that this study was single-center and had a small sample size. While the findings in our study are promising, the relatively small sample size limits the generalizability of our conclusions at this stage. Biomarker-guided risk assessment could indeed offer a cost-effective and less time-consuming alternative, particularly for resource-limited settings. However, further large-scale studies are required to validate this approach and establish its reliability in comparison to imaging-based methods like persistent LGE. We believe that this line of inquiry holds great potential and could pave the way for more accessible and economically feasible strategies in the future. Therefore, our results should be confirmed by multicenter studies with larger sample sizes. The third limitation is the lack of use of feature tracking methods to analyze regional heart deformation. Instead, we utilized CMR imaging techniques, including T1 and T2 mapping as well as LGE evaluation, as primary tools to assess myocardial changes and parameters indicative of heart deformation. We acknowledge the potential of feature tracking in providing detailed insights into regional heart deformation and consider it a valuable area for future research. The fourth limitation is that EMB was not performed as the gold standard to define myocarditis in this study. Clinical criteria were used for the diagnosis of myocarditis.

## 5. Conclusions

We found that the majority of pediatric myocarditis patients continued to have persistent LGE on CMR after a median follow-up of 6 months. Baseline troponin, CRP, CK-MB, procalcitonin, T1 relaxation time, T2 relaxation time, and ECV were observed to be associated with persistent LGE. Since it is important to identify predictors of persistent LGE, which is associated with worse cardiovascular outcomes, in close follow-up of these patients, our study results need to be confirmed in multicenter studies with large sample sizes.

## Figures and Tables

**Table 1:** Patients’ demographic and clinical data at presentation (n = 28).

Demographic Data	Statistics
Gender	
Male	27 (96.4%)
Female	1 (3.6%)
Age, years, Median (Min-Max)	16 (9–18)
Weight (Mean ± SD)	65.6 kg (±15.2 kg)
Height (Mean ± SD)	169 cm (±9.2 cm)
Body Mass Index (Mean ± SD)	22.7 (±3.8)
**Cardiac Symptoms**	
Chest pain	26 (92.9%)
Palpitations	8 (28.6%)
Syncope	2 (7.1%)
Fatigue	11 (39.3%)
**Electrocardiogram Findings**	
Normal	14 (50%)
ST Elevation	7 (25%)
ST Depression	2 (7.1%)
Low Voltage	2 (7.1%)
Ventricular Extrasystole	1 (3.6%)
Non-Specific ST-T Changes	2 (7.1%)
**Time Intervals (Days)**	
Onset of Symptoms to Hospital Admission, day	0.5 (0–7)
Hospital Admission to Initial, day, CMR	5 (0–34)
Troponin Normalization Time, day	6.8 (2–10)
**Laboratory Results**	
Troponin T (admission) ng/L	375 (16–2694)
Admission CRP (mg/L)	19.6 (1–173)
Admission PCT (ng/mL)	0.23 (0.02–2.5)
Admission CK-MB (ng/mL)	19.6 (2–163)
**Echocardiogram Findings (Admission)**	
Normal	22 (78.6%)
Pericardial Effusion	2 (7.1%)
Reduced Systolic Functions	2 (7.1%)
Left Ventricular Dilation	1 (3.6%)
Congenital Heart Disease	1 (3.6%)
**Systolic Functions in Admission Echocardiography**	
Normal	26 (92.9%)
Reduced (SF < 28%)	2 (7.1%)
Systolic Functions in Admission Echocardiography	n (%)
Normal	26 (92.9%)
**Discharge Echocardiography EF%**	
Ejection Fraction	70 (58-74)
**Diagnostic Procedures**	
Diagnostic Cardiac Catheterization	1 (3.6%)
Normal Coronary Arteries	1
Coronary CT Angiography	5 (17.9%)
Normal	2
Aortic Root Dilation	1
Myocardial Bridging	1
Cardiomegaly	1
Viral Panel Study	21 (75%)
Negative	17 (60.7%)
Positive	4 (14.3%)
Coxsackie Virus	1
COVID-19 Virus	1
Influenza Virus	2
**Treatment Groups**	
NSAID	24 (85.7%)
Steroid	1 (3.6%)
Inotropic Therapy	1 (3.6%)
Intravenous Immunoglobulin	1 (3.6%)
Antiarrhythmic Therapy	3 (10.7%)
No Treatment Initiated	3 (10.7%)

**BAV:** bicuspid aortic valve, **CK-MB:** creatine kinase-MB (U/L), **CMR:** cardiac magnetic resonance, **CRP:** C-reactive protein (mg/L), **CT:** computed tomography, **EF:** ejection fraction (%), **NSAID:** non-steroidal anti-inflammatory drugs, **PCT:** procalcitonin (ng/mL), **SD:** standard deviation, **SF:** shortening fraction (%). Values expressed as numbers, percentages (%), medians (minimum-maximum) and means±standard deviations (SD).

**Table 2:** Evaluation of cardiac magnetic resonance (CMR) parameters. (* Wilcoxon signed ranks test, ** Chi-square test, and *** Fisher’s exact test were used. Statistically significant results are highlighted in bold).

CMR Parameter	Baseline (n: 28)	Follow-Up (n: 28)	*p*
Number of segments with LGE (n), median (min–max)	3 (1–6)	2 (0–5)	<0.001 *
Positive LGE, n (%)	28 (100%)	24 (85.7%)	0.056 ***
Increase	1 (3.6%)		
No change	2 (7.1%)		
Decrease	21 (75%)		
Normalization	4 (14.3%)		
Increased T1 times, n (%)	15 (53.6%)	7 (25%)	0.054 **
T1 relaxation time, native (ms), median (min–max)	1058 (916–1158)	1004 (942–1078)	0.001 *
ECV increase, n (%)	14 (50%)	5 (17.9%)	0.023 **
ECV fraction (%), median (min–max)	25% (20–45%)	22.5% (19–38%)	0.002 *
Increased T2 times, n (%)	17 (60.7%)	0 (0%)	<0.001 **
STIR T2 intensity area n (%)	14 (50%)	0 (0%)	<0.001 **
T2 relaxation time (ms), median (min–max)	55.5 (42–62)	46 (41–50)	<0.001 *
LVEF (%), median (min–max)	60 (14–67)	60 (32–67)	0.009 *
LV systolic dysfunction, n %	4 (14.3%)	2 (7.1%)	0.335 **
LVEDVi (mL/m^2^), median (min–max)	75(60–203)	76 (60–188)	0.939 *
LVESVi (mL/m^2^), median (min–max)	30 (22–169)	30 (21–126)	0.139 *
Pericardial effusion, n%	4 (14.3%)	0 (0%)	0.056 ***

Abbreviations: CMR: cardiac magnetic resonance, ECV: extracellular volume (%), LGE: late gadolinium enhancement, LV: left ventricle, LVEDVi: left ventricular end-diastolic volume index (mL/m²), LVEF: left ventricular ejection fraction (%), LVESVi: left ventricular end-systolic volume index (mL/m²), STIR: short tau inversion recovery.

**Table 3:** Comparison of patients with persistent late gadolinium enhancement (LGE) and those with resolving LGE on cardiac magnetic resonance imaging.

Parameters		LGE Persistence	LGE Resolution	*p*
		(n: 24)	(n: 4)	
Gender	Male	23 (%95.8)	4 (%100)	0.857 *
	Female	1 (%4.2)	0 (%0)	
Troponin T ng/L		496 (243–1175)	106.5 (26.5–299)	0.022 **
CRP mg/dL		24.4 (14.3–71.5)	2.98 (1.16–4.51)	0.016 **
Initial CMR T1 Relaxation Time (ms)		1072 (1010–1103)	960 (929–987)	0.009 **
Initial CMR ECV Increase (%)		28 (22–33.5)	21 (20.2–22.5)	0.029 **
Initial CMR T2 Relaxation Time (ms)		56.5 (48–59.75)	46 (44.25–47.75)	0.032 **
Initial CMR LGE Segment Count		3.5 (3–5)	2.5 (1.25–3.75)	0.097 **
Initial CMR LVEF (%)		59.5 (58–62.25)	60.5 (60–61.75)	0.230 **

Variables marked with an asterisk (*) were analyzed using the Chi-square test, and those marked with two asterisks (**) were analyzed using the Mann–Whitney U test. Values are presented as n (%) for categorical variables or as median (25th–75th quartiles) for continuous variables. Statistically significant results are highlighted in bold. Abbreviations: CMR: cardiac magnetic resonance, CRP: C-reactive protein (mg/L), LGE: late gadolinium enhancement, LVEF: left ventricular ejection fraction (%).

**Table 4:** Relationship of T1, T2, and extracellular volume values with biochemical parameters in the baseline cardiac magnetic resonance evaluation.

	T1 Relaxation Times			T2 Relaxation Times			Extracellular Volume		
	Increase	No Change	*p*	Increase	No Change	*p*	Increase	No Change	*p*
**Troponin T ng/L**	1032 (71–2694)	339 (16–1568)	0.053	1032 (281–1375	155 (79–462)	**0.011**	1048 (281–1375)	288 (103–605)	**0.020**
**Troponin-T normalization (days)**	7.7 (6.6–8.7)	6 (4.5–7.5)	**0.043**	7.7 (6.5–8.7)	5.7 (4.2–7.2)	**0.021**	8 (6.7–9)	6 (4.7–7.2)	**0.041**
**Procalcitonin ng/mL**	0.38 (0.19–0.77)	0.14 (0.07–0.27)	**0.024**	0.21 (0.02–1)	0.25 (0.08–2.5)	0.981	0.37 (0.02–1)	0.17 (0.02–2.5)	0.102
**CK-MB (ng/mL)**	29 (2–163)	12 (2–57)	0.134	25.4 (3–163)	8.87(2–57)	0.051	37.5 (13.4–66.4)	10.4 (4.2–28.4)	**0.039**

Statistically significant results are highlighted in bold. Abbreviations: CK-MB: creatine kinase-MB (U/L).

## Data Availability

The data that support the findings of this study are available from the corresponding author upon reasonable request.

## References

[1] Law Y.M., Lal A.K., Chen S., Cihakova D., Cooper L.T., Deshpande S., Godown J., Grosse-Wortmann L., Robinson J.D., Towbin J.A. (2021). Diagnosis and Management of Myocarditis in Children: A Scientific Statement From the American Heart Association. Circulation.

[2] Vasudeva R., Bhatt P., Lilje C., Desai P., Amponsah J., Umscheid J., Parmar N., Bhatt N., Adupa R., Pagad S. (2021). Trends in Acute Myocarditis Related Pediatric Hospitalizations in the United States, 2007–2016. Am. J. Cardiol..

[3] Williams J.L., Jacobs H.M., Lee S. (2023). Pediatric Myocarditis. Cardiol Ther..

[4] Fabre A., Sheppard M.N. (2006). Sudden adult death syndrome and other non-ischaemic causes of sudden cardiac death. Heart.

[5] Maron B.J., Doerer J.J., Haas T.S., Tierney D.M., Mueller F.O. (2009). Sudden deaths in young competitive athletes: Analysis of 1866 deaths in the United States, 1980–2006. Circulation.

[6] Harris K.M., Mackey-Bojack S., Bennett M., Nwaudo D., Duncanson E., Maron B.J. (2021). Sudden Unexpected Death Due to Myocarditis in Young People, Including Athletes. Am. J. Cardiol..

[7] Khanal S., Khanal B., Chou F.-S., Moon-Grady A.J., Ghimire L.V. (2024). Comparison of mortality and cardiovascular complications due to COVID-19, RSV, and influenza in hospitalized children and young adults. BMC Cardiovasc. Disord..

[8] Sozzi F.B., Gherbesi E., Faggiano A., Gnan E., Maruccio A., Schiavone M., Iacuzio L., Carugo S. (2022). Viral Myocarditis: Classification, Diagnosis, and Clinical Implications. Front. Cardiovasc. Med..

[9] Butts R.J., Boyle G.J., Deshpande S.R., Gambetta K., Knecht K.R., Prada-Ruiz C.A., Richmond M.E., West S.C., Lal A.K. (2017). Characteristics of Clinically Diagnosed Pediatric Myocarditis in a Contemporary Multi-Center Cohort. Pediatr. Cardiol..

[10] Messroghli D.R., Moon J.C., Ferreira V.M., Grosse-Wortmann L., He T., Kellman P., Mascherbauer J., Nezafat R., Salerno M., Schelbert E.B. (2017). Clinical recommendations for cardiovascular magnetic resonance mapping of T1, T2, T2* and extracellular volume: A consensus statement by the Society for Cardiovascular Magnetic Resonance (SCMR) endorsed by the European Association for Cardiovascular Imaging (EACVI). J. Cardiovasc. Magn. Reson..

[11] Ali-Ahmed F., Dalgaard F., Al-Khatib S.M. (2020). Sudden cardiac death in patients with myocarditis: Evaluation, risk stratification, and management. Am. Heart J..

[12] Peretto G., Sala S., Rizzo S., Palmisano A., Esposito A., De Cobelli F., Campochiaro C., De Luca G., Foppoli L., Dagna L. (2020). Ventricular Arrhythmias in Myocarditis: Characterization and Relationships With Myocardial Inflammation. J. Am. Coll. Cardiol..

[13] Ferreira V.M., Schulz-Menger J., Holmvang G., Kramer C.M., Carbone I., Sechtem U., Kindermann I., Gutberlet M., Cooper L.T., Liu P. (2018). Cardiovascular Magnetic Resonance in Nonischemic Myocardial Inflammation: Expert Recommendations. J. Am. Coll. Cardiol..

[14] Banka P., Robinson J.D., Uppu S.C., Harris M.A., Hasbani K., Lai W.W., Richmond M.E., Fratz S., Jain S., Johnson T.R. (2015). Cardiovascular magnetic resonance techniques and findings in children with myocarditis: A multicenter retrospective study. J. Cardiovasc. Magn. Reson..

[15] Raimondi F., Iserin F., Raisky O., Laux D., Bajolle F., Boudjemline Y., Boddaert N., Bonnet D. (2015). Myocardial inflammation on cardiovascular magnetic resonance predicts left ventricular function recovery in children with recent dilated cardiomyopathy. Eur. Heart J.-Cardiovasc. Imaging.

[16] Cornicelli M.D., Rigsby C.K., Rychlik K., Pahl E., Robinson J.D. (2019). Diagnostic performance of cardiovascular magnetic resonance native T1 and T2 mapping in pediatric patients with acute myocarditis. J. Cardiovasc. Magn. Reson..

[17] Di Lisi D., Madaudo C., Carmina M.G., Clemenza F., Scelfo D., La Franca E., Pieri M., Vitale G., Galassi A.R., Novo G. (2024). Prognosis of myocarditis stratified by initial clinical presentation: Does “intermediate” risk still play a role?. Am. Heart J. Plus..

[18] Cerqueira M.D., Weissman N.J., Dilsizian V., Jacobs A.K., Kaul S., Laskey W.K., Pennell D.J., Rumberger J.A., Ryan T., Verani M.S. (2002). Standardized myocardial segmentation and nomenclature for tomographic imaging of the heart. A statement for healthcare professionals from the Cardiac Imaging Committee of the Council on Clinical Cardiology of the American Heart Association. Circulation.

[19] Richardson P., McKenna W., Bristow M., Maisch B., Mautner B., O’Connell J., Olsen E., Thiene G., Goodwin J., Gyarfas I. (1996). Report of the 1995 World Health Organization/International Society and Federation of Cardiology Task Force on the Definition and Classification of cardiomyopathies. Circulation.

[20] Friedrich M.G., Sechtem U., Schulz-Menger J., Holmvang G., Alakija P., Cooper L.T., White J.A., Abdel-Aty H., Gutberlet M., Prasad S. (2009). Cardiovascular magnetic resonance in myocarditis: A JACC white paper. J. Am. Coll. Cardiol..

[21] Kawel-Boehm N., Hetzel S.J., Ambale-Venkatesh B., Captur G., Francois C.J., Jerosch-Herold M., Salerno M., Teague S.D., Valsangiacomo-Buechel E., van der Geest R.J. (2020). Reference ranges (“normal values”) for cardiovascular magnetic resonance (CMR) in adults and children: 2020 update. J. Cardiovasc. Magn. Reson..

[22] Yang F., Wang J., Li W., Xu Y., Wan K., Zeng R., Chen Y. (2020). The prognostic value of late gadolinium enhancement in myocarditis and clinically suspected myocarditis: Systematic review and meta-analysis. Eur. Radiol..

[23] Isaak A., Bischoff L.M., Faron A., Endler C., Mesropyan N., Sprinkart A.M., Pieper C.C., Kuetting D., Dabir D., Attenberger U. (2021). Multiparametric cardiac magnetic resonance imaging in pediatric and adolescent patients with acute myocarditis. Pediatr. Radiol..

[24] Luetkens J.A., Homsi R., Dabir D., Kuetting D.L., Marx C., Doerner J., Schlesinger-Irsch U., Andrié R., Sprinkart A.M., Schmeel F.C. (2016). Comprehensive Cardiac Magnetic Resonance for Short-Term Follow-Up in Acute Myocarditis. J. Am. Heart Assoc..

[25] Schauer J., Buddhe S., Gulhane A., Sagiv E., Studer M., Colyer J., Chikkabyrappa S.M., Law Y., Portman M.A. (2022). Persistent cardiac magnetic resonance imaging findings in a cohort of adolescents with post-coronavirus disease 2019 mRNA vaccine myopericarditis. J. Pediatr..

[26] Dubey S., Agarwal A., Nguyen S., Adebo D. (2020). Persistence of late gadolinium enhancement on follow-up CMR imaging in children with acute myocarditis. Pediatr. Cardiol..

[27] Lota A.S., Tsao A., Owen R., Halliday B.P., Auger D., Vassiliou V.S., Tayal U., Almogheer B., Vilches S., Al-Balah A. (2021). Prognostic significance of nonischemic myocardial fibrosis in patients with normal LV volumes and ejection-fraction. Cardiovasc. Imaging.

[28] Berg J., Kottwitz J., Baltensperger N., Kissel C.K., Lovrinovic M., Mehra T., Scherff F., Schmied C., Templin C., Lüscher T.F. (2017). Cardiac Magnetic Resonance Imaging in Myocarditis Reveals Persistent Disease Activity Despite Normalization of Cardiac Enzymes and Inflammatory Parameters at 3-Month Follow-Up. Circ. Heart Fail..

[29] Mahrholdt H., Wagner A., Deluigi C.C., Kispert E., Hager S., Meinhardt G., Vogelsberg H., Fritz P., Dippon J., Bock C.-T. (2006). Presentation, patterns of myocardial damage, and clinical course of viral myocarditis. Circulation.

[30] White J.A., Hansen R., Abdelhaleem A., Mikami Y., Peng M., Rivest S., Satriano A., Dykstra S., Flewitt J., Heydari B. (2019). Natural History of Myocardial Injury and Chamber Remodeling in Acute Myocarditis. Circ. Cardiovasc. Imaging.

[31] Pieroni M., Ciabatti M., Zocchi C., Tavanti V., Camporeale A., Saletti E., Fumagalli C., Venezia D., Lombardi M., Olivotto I. (2024). Optimal timing of follow-up cardiac magnetic resonance in patients with uncomplicated acute myocarditis. Int. J. Cardiol..

[32] Małek Ł.A., Kamińska H., Barczuk-Falęcka M., Ferreira V.M., Wójcicka J., Brzewski M., Werner B. (2020). Children with acute myocarditis often have persistent subclinical changes as revealed by cardiac magnetic resonance. J. Magn. Reson. Imaging.

[33] Aquaro G.D., Habtemicael Y.G., Camastra G., Monti L., Dellegrottaglie S., Moro C., Lanzillo C., Scatteia A., Di Roma M., Pontone G. (2019). Prognostic Value of Repeating Cardiac Magnetic Resonance in Patients With Acute Myocarditis. J. Am. Coll. Cardiol..

[34] Sachdeva S., Song X., Dham N., Heath D.M., DeBiasi R.L. (2015). Analysis of clinical parameters and cardiac magnetic resonance imaging as predictors of outcome in pediatric myocarditis. Am. J. Cardiol..

[35] Mewton N., Dernis A., Bresson D., Zouaghi O., Croisille P., Flocard E., Douek P., Bonnefoy-Cudraz E. (2015). Myocardial biomarkers and delayed enhanced cardiac magnetic resonance relationship in clinically suspected myocarditis and insight on clinical outcome. J. Cardiovasc. Med. (Hager-Stown).

[36] Grun S., Schumm J., Greulich S., Wagner A., Schneider S., Bruder O., Kispert E.-W., Hill S., Ong P., Klingel K. (2012). Long-term follow-up of biopsy-proven viral myocarditis: Predictors of mortality and incomplete recovery. J. Am. Coll. Cardiol..

[37] Ferrero P., Piazza I. (2020). QRS fragmentation in children with suspected myocarditis: A possible additional diagnostic sign. Cardiol. Young.

[38] Chong D., Chua Y.T., Chong S.L., Ong G.Y. (2018). What Raises Troponins in the Paediatric Population?. Pediatr Cardiol..

